# Mental well-being of healthcare workers in two hospital districts with differing COVID-19 incidence: Noora Rantanen

**DOI:** 10.1093/eurpub/ckac130.145

**Published:** 2022-10-25

**Authors:** N Rantanen, J Lieslehto, L-MAH Oksanen, SA Oksanen, V-J Anttila, L Lehtonen, A Geneid, E Sanmark

**Affiliations:** 1 Faculty of Medicine, University of Helsinki, Helsingin yliopisto, Finland; 2 Clinical Research Institute HUCH, Helsinki University Hospital, Helsinki, Finland; 3 Niuvanniemi Hospital, University of Eastern Finland, Kuopio, Finland; 4 Department of Otorhinolaryngology and Phoniatrics, Helsinki University Hospital, Helsinki, Finland; 5 BeeHealthy, Helsinki, Finland; 6 HUS Inflammation Center, Helsinki University Hospital, Helsinki, Finland; 7 HUS Diagnostic Center, HUSLAB, Helsinki University Hospital, Helsinki, Finland

## Abstract

**Objectives:**

Healthcare systems and healthcare workers have been under considerable strain during the COVID-19 pandemic in many countries. Our study aimed to assess the mental well-being of Finnish healthcare workers from two hospital districts with differing COVID-19 incidence rates (HUS, Hospital district of Helsinki and Uusimaa/Helsinki University Hospital; and Kymsote, Social and Health services in Kymenlaakso region) during the first wave of the COVID-19 pandemic in spring 2020.

**Material and methods:**

The data of this prospective survey study was collected during summer 2020, and a total of 996 healthcare workers (HUS N = 862, Kymsote N = 134) participated. Mental health symptoms were self-reported, and symptom criteria followed ICD-10 classification, excluding duration criteria. We divided participants into symptom categories “often/sometimes” (those who met the diagnostic criteria), and “rarely/never” (those not meeting the diagnostic criteria), and compared these groups to sociodemographic factors and factors related to work, workload, and well-being.

**Results:**

Despite differences in COVID-19 incidence, the degree of mental health symptoms did not differ between HUS and Kymsote districts (p = 1). A significant relationship was found between self-reported diagnostic mental health symptoms and experiences of insufficient instructions for protection against COVID-19 (in HUS cohort, p < 0.001), insufficient recovery from work (p < 0.001), and subjective increased workload (p < 0.001).

**Conclusions:**

These findings show the importance of sufficient, well-designed instructions for protection from SARS-CoV-2 for healthcare workers, indicating their need to feel safe and protected at work. The workload of healthcare workers should be carefully monitored to keep it moderate and ensure their adequate recovery from work. Sufficient control of the epidemic to keep the burden of the healthcare system as low as possible is essential for healthcare workers’ well-being.

**Key messages:**

- Workload of healthcare workers should be carefully monitored during a pandemic to keep it moderate and ensure adequate recovery in all regions, regardless of the local disease burden.
- The importance of sufficient, well-designed instructions for healthcare workers and their need to feel safe and protected at work.

